# Carbon Dot-Triggered Photocatalytic Degradation of Cellulose Acetate

**DOI:** 10.1021/acs.biomac.1c00273

**Published:** 2021-04-27

**Authors:** Nisha Yadav, Karin H. Adolfsson, Minna Hakkarainen

**Affiliations:** † Department of Fibre and Polymer Technology, KTH Royal Institute of Technology, Teknikringen 56, Stockholm 100 44, Sweden; ‡ Wallenberg Wood Science Center (WWSC), KTH Royal Institute of Technology, Teknikringen 56, Stockholm 100 44, Sweden

## Abstract

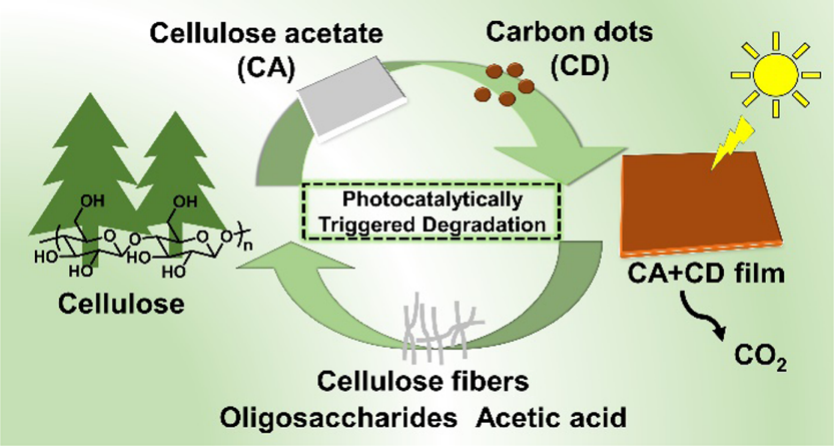

Chemical modification of biopolymers, before use in thermoplastic applications, can reduce the susceptibility to open environment degradation. We demonstrate carbon dots (CDs) as green photocatalytic triggers that can render the common cellulose derivative, cellulose acetate (CA), degradable under open environment relevant conditions. CD-modified cellulose acetate (CA + CD) films were subjected to UV-A irradiation in air and simulated sea water, and the degradation process was mapped by multiple spectroscopic, chromatographic, and microscopy techniques. The addition of CDs effectively catalyzed the deacetylation reaction, the bottleneck preventing biodegradation of CA. The photocatalytically activated degradation process led to significant weight loss, release of small molecules, and regeneration of cellulose fibers. The weight loss of CA + CD after 30 days of UV-A irradiation in air or simulated sea water was 53 and 43%, respectively, while the corresponding values for plain CA films were 12 and 4%. At the same time the weight average molar mass of CA + CD decreased from 62,000 to 11,000 g/mol and 15,000 g/mol during UV-A irradiation in air and simulated sea water, respectively, and the degree of substitution (DS) decreased from 2.2 to 1.6 both in air and in water. The aging in water alone did not affect the weight average molar mass, but the DS was decreased to 1.9. Control experiments confirmed the generation of hydrogen peroxide when aqueous CD dispersion was subjected to UV-A irradiation, indicating a free radical mechanism. These results are promising for the development of products, such as mulching films, with photocatalytically triggered environmental degradation processes.

## Introduction

Every year, millions of tons of plastics are released to our oceans resulting in potentially major damage to marine life and human health.^1,2^ Multiple approaches are urgently required to improve the situation, from reduce and reuse of plastics to effective waste management, design for recycling, and in some cases design for open environment degradation.^3,4^ Green and effective photocatalytic triggers could be a benign route to plastics and bioplastics with susceptibility to open environment degradation or chemical recyclability under mild conditions.^5,6^ Cellulose is a natural biopolymer and inherently degradable in suitable natural environments but the chemical modification that is needed for gaining thermoplastic properties and hydrophobicity ultimately reduces its susceptibility to environmental degradation.^7,8^ Cellulose acetate (CA) is the most common commercial cellulose derivative for thermoplastic applications.^9^ It is typically used for production of cigarette filters, textiles, and packaging. Among plastic litter, CA in the form of cigarette butts (CBs) is one of the most common types of littered item with an estimated 4.5 trillion CBs discarded annually.^10^ This represents 22–46% of visible litter in urban areas.^11^ Unfortunately, although CA is a derivative of cellulose, it can persist in the environment for more than 10 years depending on the location and environmental conditions.^12−15^

According to the existing literature, CA is potentially biodegradable,^16,17^ but the biodegradability highly depends on the degree of substitution (DS) by acetylation. According to Gardner et al., as the DS increases, biodegradability decreases or is entirely halted.^18^ Exposure of ultraviolet light (UV light) from the sun has the ability to physically break down many plastics into small pieces by generating free radical species and follow-up oxidative reactions leading to release of CO_2_ and other small molecules.^19^ The photodegradation of cellulose, CA, and cellulose triacetate have been investigated using irradiation at a wavelength of 275 nm.^20−22^ Deacetylation was not apparent in the case of cellulose triacetate, but chain scission, oxidation, and cross-linking were observed in the presence of oxygen. The absorption maximum for CA is at ∼260 nm, while the cutoff for sunlight reaching earth is at 300 nm. This indicates that pure CA might not be significantly degraded by natural sunlight. However, photocatalysts or photosensitizers can act as chromophores and initiate photodegradation of CA. Different photosensitizers such as benzophenone, acetophenone, 4,4′-bis(dimethylamino)benzophenone, triphenylsulphonium trifluoromethanesulfonate (TPS), and diphenyliodonium trifluoromethanesulfonate (DPI) have been shown to accelerate the photodegradation of the CA polymer.^23,24^ CA films were also modified with titanium dioxide (TiO_2_) to increase the susceptibility to UV irradiation. This addition further enhanced the enzymatic hydrolysis rate by cellulase enzyme due to the increased hydrophilicity and surface area and lowered the DS and zeta potential.^25−27^ It was further shown that TiO_2_ nanoparticles embedded into CA fibers significantly accelerated the degradation of CA fibers.^26,28^ Anatase pigments, the surface of which had been treated with Ba/Ca sulphates or phosphates, were also used, with the aim of further increasing the photocatalytic effect. However, an aggregation issue with TiO_2_ nanoparticles led to heterogeneous distribution and decreased photocatalytic efficiency, which resulted in only moderately accelerated degradation rates.^29−31^ To prevent agglomeration and to improve the distribution, surface functionalization or metal/nonmetal doping of TiO_2_ was evaluated. However, these modifications typically required high temperatures and multistep experimental processes. A material modification by photocatalytically active carbon-modified TiO_2_ resulted in up to ∼32% weight loss in an outdoor environment.^32,33^

The high degree of acetylation is the bottleneck for biodegradation or open environment degradation of CA. Finding safe and effective triggers or catalysts to facilitate the deacetylation process is therefore of high interest. Photoactive carbon dots (CDs) could have high potential for catalyzing such reactions under the influence of sunlight. In earlier studies we demonstrated that CDs could improve multiple material properties and processability of poly(caprolactone) (PCL), poly(lactide) (PLA), and starch.^34−36^ CDs are also generally regarded as environmentally benign materials with good biocompatibility.^37^

CDs can photocatalytically degrade dyes under visible light.^38^ Therefore, we hypothesized that modification of CA with CDs could trigger the degradation of CA under sunlight by catalyzing deacetylation. To examine this hypothesis, we produced CDs by microwave-assisted hydrothermal carbonization (HTC) and oxidation of α-cellulose and fabricated CA films with and without CDs. The susceptibility of the materials to degradation under UV-A irradiation (simulated sunlight) in air or simulated sea water was evaluated at the molecular level by characterizing the matrix changes and released products by multiple spectroscopic, chromatographic, microscopy, and thermal analysis techniques.

## Experimental Section

### Materials

α-Cellulose, sulfuric acid (H_2_SO_4_; 95–98%), nitric acid (70%; HNO_3_), cellulose acetate (CA; 30 KDa), H_2_O_2_ (30 wt%), 2,9-Dimethyl-1,10-Phenanthroline (DMP) and copper(II) sulfate were obtained from Sigma-Aldrich. Sodium hydroxide pellets were purchased from Merck. Acetone and dimethyl sulfoxide (DMSO) were of technical grade and purchased from VWR. Preparation of simulated sea water was done according to ASTM standard D6691–17 without the presence of any microorganisms rendering to average salinity of world sea water. A phosphate buffer solution (0.1 M) was prepared from K_2_HPO_4_ and NaH_2_PO_4_ (Sigma-Aldrich) with pH adjusted to 7.0 by H_2_SO_4_ (1 N, VWR) and NaOH (1 N, VWR). All chemicals were used as-received.

### Synthesis of Nano-Graphene Oxide-Type CDs

The synthesis of CDs was performed according to our previously published work through microwave-assisted HTC of cellulose with some modifications.^37^ Briefly, in a Teflon vessel, 2 g of α-cellulose and 20 mL of 0.1 g/mL H_2_SO_4_ were added. Carbonization was performed in a flexiWAVE microwave (Milestone Inc.) with the following program: the temperature was raised to 220 °C with a ramp time of 20 min and then kept at 220 °C for 2 h with addition of stirring inside the vessels. The temperature was monitored with a probe inside one of the vessels. After the reaction, the resulting black carbon spheres (CSs) were filtered, washed with water, and dried in a vacuum oven for 24 h. CSs (250 mg) were then added in 25 mL of 70% HNO_3_ in a round-bottom flask and left to sonicate for 1 h at 45 °C. The reaction mixture was then refluxed in an oil bath at 90 °C for 30 min, after which it was poured into 200 mL of H_2_O, and the acidic water with HNO_3_ was evaporated with a rotary evaporator. The final product was vacuum-dried and the obtained oxidized material, nano-graphene oxide (nGO) type CDs, was reddish in color.

### Preparation of CA and CA + CD Films

Solution casting was utilized for the preparation of CA + CD composites. CA (30 mg/mL) and additional 2.5 wt % of CDs were dissolved in 5 mL acetone. The dispersions were then sonicated for 5 min in a sonication bath (Bransonic ultrasonic cleaner, model 2210) and cast on dust-free Teflon molds (6 cm × 6 cm × 6 cm) and dried in an oven at 80 °C. All films were kept in desiccators for further characterizations. The films were abbreviated as CA and CA + CD, for the plain CA and CD-containing films, respectively. The average thickness of the films was 75 ± 5 μm. The thickness of the films was measured at five different locations using a MITUTOYO CORP ID-C112TB absolute digital gauge instrument and reported as the average of the five measurements.

### 2,9-Dimethyl-1,10-Phenanthroline (DMP) Method

A procedure as described by Kosaka et al.^39^ and Baga et al.^40^ was adopted with some modification. One milliliter each of DMP, copper(II) sulfate, and phosphate buffer (pH 7.0) was added to a 10 mL volumetric flask and mixed. To detect the formation of H_2_O_2_, a measured volume of CD dispersion was added to the volumetric flask and then the flask was filled up with deionized water. To prepare the calibration curve, a measured volume of H_2_O_2_ solution was added instead. After mixing, the absorbance of the sample or calibration solution (at 454 nm) was measured. A blank solution was prepared in the same manner but without H_2_O_2_ and CDs. Using the difference in absorbance between the H_2_O_2_ solutions and blank solution, a calibration curve was generated.

From the calibration curve, H_2_O_2_ concentrations in the CD samples were calculated as follows:
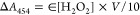
1where Δ*A*_454_ is the difference of the absorbance between the sample and blank solutions at 454 nm, ϵ is the slope of the calibration curve, [H_2_O_2_] is the H_2_O_2_ concentration (μM), and *V* is the sample volume (mL).

### Description of the Samples for the Degradation Experiments

The samples for degradation experiments were set up in triplicate for each sample type (CA and CA + CD) and each environment. The aging time was 30 days. The samples were rectangular strips of films, 5 cm × 0.5 cm × 75 μm, and abbreviated according to sample type and aging conditions.

**(a) CA**: Neat CA film before degradation.
**(b) CA + CD**: CA + CD film before degradation.
**(c) CA-UV**: Neat CA film exposed to UV radiation.
**(d) CA + CD-UV**: CA + CD film exposed to UV radiation.
**(e) CA-Sea water (CA-SW)**: Neat CA film dipped in 20 mL of ASTM standard laboratory synthesized simulated sea water in Teflon septa-sealed glass vials on a shaker (speed 200 rpm).
**(f) CA + CD-Sea water (CA + CD-SW)**: CA + CD film dipped in 20 mL of ASTM standard laboratory synthesized simulated sea water in Teflon septa-sealed glass vials on a shaker (speed 200 rpm).
**(g) CA-Sea water-UV (CA-SW-UV)**: Neat CA film dipped in 20 mL of ASTM standard laboratory synthesized simulated sea water in Teflon septa-sealed glass vials under UV irradiation.
**(h) CA + CD-Sea water-UV (CA + CD-SW-UV)**: CA + CD film dipped in 20 mL of ASTM standard laboratory synthesized simulated sea water in Teflon septa-sealed glass vials under UV irradiation.

### UV-A Irradiation

Experiments involving simulated sunlight were carried out in an G23 lamp base chamber 210 × 250 × 95 mm (L × W × H) equipped with a power supply of 240 V, 50 Hz, 36 W. The wavelength of the UV light was 370 nm simulating long wavelength UV-A radiation from the sun (∼95% of the UV radiation from the sun reaching the earth’s surface is UV-A radiation). During the UV irradiation, the temperature of the UV chamber increased to approximately 50 °C.

## Measurements and Characterization

### Fourier Transform Infrared Spectroscopy (FTIR)

Fourier transform infrared spectra of α-cellulose, CSs, CDs, CA films, and CA + CD composite films before and after degradation were obtained by a PerkinElmer Spectrum 2000 FTIR spectrometer. A total of 16 scans were recorded in the wavenumber area of 600 to 4000 cm^–1^.

### Ultraviolet–visible Spectroscopy (UV–vis)

The absorption of CA and CA + CD films were measured by a Shimadzu UV-2550 UV–vis spectrophotometer. The measurements were carried out through solid rectangular strips with a slit width of 1 cm.

### Thermal Gravimetric Analysis (TGA)

Mettler Toledo TGA/SDTA 851e was utilized for the thermogravimetric analysis of cellulose, CSs, CDs, CA films, and CA + CD composite films, before and after degradation. A total of 2–5 mg of each sample was placed into a 70 μL alumina cup. The samples were then heated at the rate of 10 °C min^–1^ from 50 to 800 °C with a N_2_ flow rate of 50 mL min^–1^.

### Differential Scanning Calorimetry (DSC)

The thermal properties of CA and CA + CD films before and after degradation were analyzed using a Mettler Toledo DSC instrument. The sample size was 2–5 mg, and the analyses were performed in a N_2_ atmosphere with a flow rate of 50 mL/min. The method was as follows: the sample was kept at 30 °C for 2 min, then heated up to 300 °C at the rate of 10 °C/min, kept at 300 °C for 2 min, and then the temperature was decreased to 30 °C at the rate of 10 °C/min, kept at 30 °C for 2 min, and then heated up again to 300 °C at the rate of 10 °C/min.

### X-ray Diffraction (XRD)

X-ray diffraction spectra were recorded for α-cellulose, CSs, CDs, CA, and CA + CD before and after degradation using the X-ray source Cu KR radiation (λ = 0.1541 nm). The diffraction was measured by a PANalytical X’Pert PRO diffractometer at 25 °C with a silicium monocrystal sample holder. The intensity was determined in a 2θ angular range between 5 and 40° with a step size of 0.017° for all analyses.

### Nuclear Magnetic Resonance Spectroscopy (NMR)

^1^H NMR spectra were recorded on a Bruker Avance 400 MHz spectrometer with 64 scans. The samples (5 mg) were dissolved in 0.7 mL deuterated dimethyl sulfoxide (DMSO-d_6_) in a 5 mm diameter NMR tube. A couple of drops of trifluoroacetic acid (TFA) were added to shift the peak of exchangeable protons downfield in the ^1^H NMR spectra. The DS for acetylation of CA was calculated from the ^1^H NMR spectra according to eq 2.

The ^1^H NMR spectrum of CA and CA exposed to different environment conditions exhibited spectral lines in the ring proton region at 5.3–2.8 ppm and for the acetyl groups at 1.80–2.15 ppm.
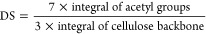
2

### Scanning Electron Microscopy (SEM)

SEM images were acquired by an ultrahigh resolution FE-SEM Hitachi S-4800. The samples were sputter-coated (Cressington 208HR Sputter Coater) with platinum/palladium (Pt/Pd) at 2 nm thickness prior to the analysis.

### Size Exclusion Chromatography (SEC)

The average molar masses (*M*_n_, *M*_w_, and *M*_z_) and dispersity (*Đ*) of CA and CA + CD before and after degradation under different environmental conditions were analyzed by SEC. The analyses were performed in DMSO/0.5 wt % LiCl at 23 °C using an Agilent size exclusion chromatograph equipped with a Knauer 2320 refractometer index detector and two PLGel columns (MIXED-D and 103A). Before analysis, the samples were dissolved in DMSO (3 mg/mL) and 20 μL of the solutions were injected into the columns using a flow rate of 1 mL/min. Monodisperse pullulan standards were used for the calibration.

## Results and Discussion

CA was modified with CDs produced by the two-step reaction of microwave-assisted HTC of α-cellulose and oxidation of the carbonized products. The potential of CD modification to photocatalytically trigger the degradation of CA was evaluated by subjecting neat CA and CD-modified CA films to simulated sunlight (UV-A radiation) in air and simulated sea water. For comparison, the degradation process in simulated sea water without UV radiation was also investigated.

### Synthesis and Characterization of CDs

**Figure 1:**
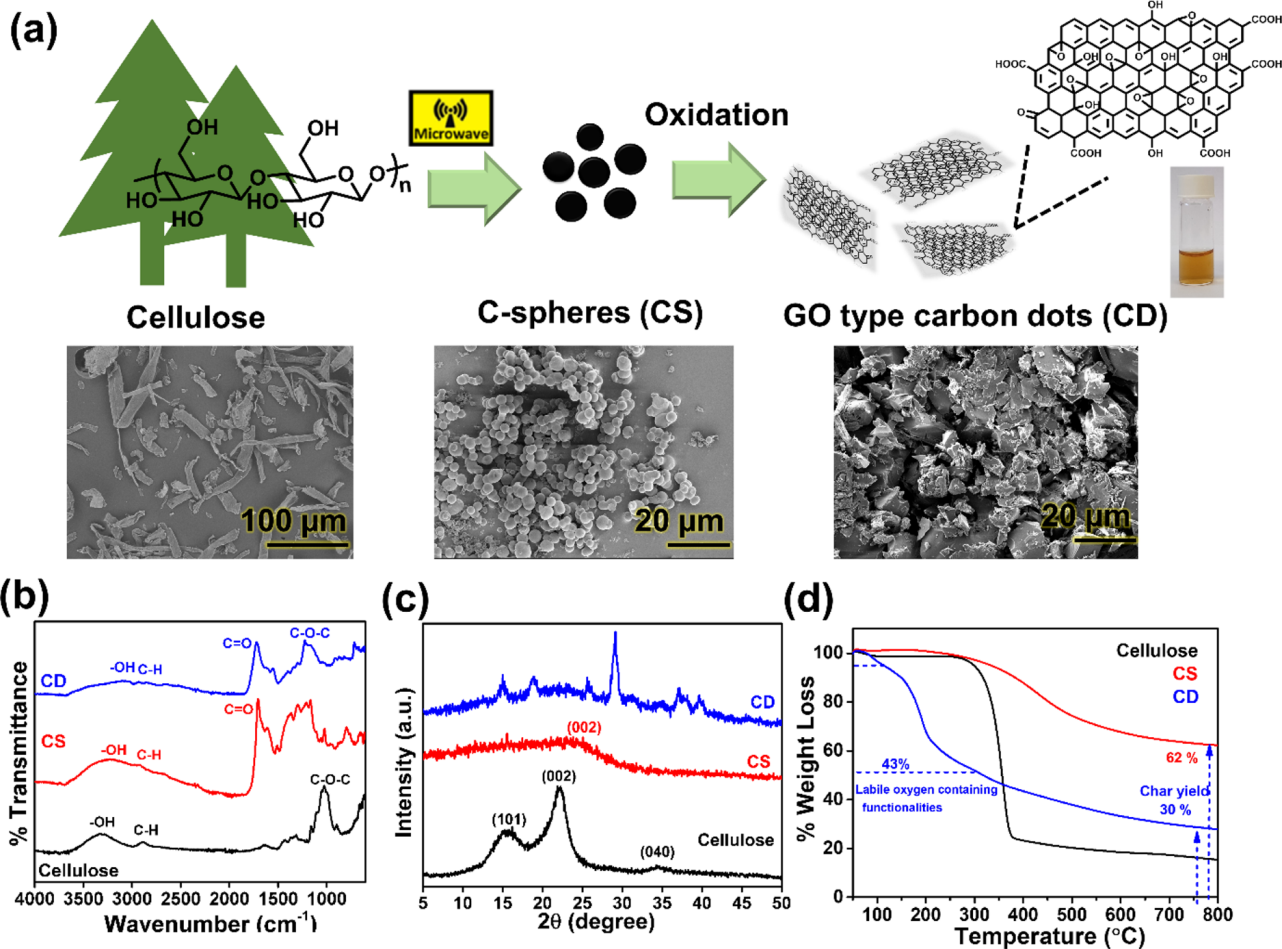
(a) Schematic presentation of the two-step synthesis of CDs via HTC and subsequent oxidation including representative SEM images of α-cellulose, the CS intermediate, and the final CD product. Characterizations of α-cellulose, CSs, and CDs by (b) FTIR, (c) powder XRD, and (d) TGA.

CDs were synthesized by microwave-assisted HTC of cellulose followed by oxidation according to our previous work (Figure 1a).^37^ In the HTC process, the cellulose fibers were carbonized to black CSs and subsequently oxidized to nGO-type CDs. The synthesized CDs were a reddish powder with good water dispersibility. The successful formation of CDs was confirmed through spectroscopic methods. The FTIR spectrum of cellulose showed the characteristic peaks at 3300, 2980, and 1050 cm^–1^ corresponding to OH, C–H, and C–O stretches, respectively (Figure 1b), whereas the FTIR spectra of the produced particles CSs and CDs showed a broad peak of unbound H_2_O, C–OH, and C–H stretches at 2500–3500 cm^–1^ and 1230 cm^–1^.^41^ Additional functionalities of CSs and CDs were the C=O stretch at 1720 cm^–1^, C=C stretch at 1618 cm^–1^, and C–O stretch at 1228 cm^–1^. The spectrum of CDs showed further an absorption band at 1540 cm^–1^, indicating organic nitrogen originating from the oxidation with HNO_3_.^42^

The XRD spectrum of cellulose showed the crystalline planes [101], [002], and [040] as in cellulose I at 2θ = 16, 22.5, and 34.8°, respectively (Figure 1c). Following the HTC process, the crystalline structure of cellulose was disrupted and the spectrum of CSs illustrated a broad peak at ∼22° belonging to the [002] plane. The [002] reflection of carbonized materials has been ascribed to the graphitic plane.^43^ After oxidation of CSs to CDs, a polydisperse crystal structure was observed, which could originate from the assembly of multilayer structures in the solid state.^44^ The peaks around 15, 19, 26, and 29° may stem for the additional functional oxygen groups in the CD, but they could also be caused by inorganic salts originating from H_2_SO_4_ or HNO_3_.^37,45^Figure 1d presents the TGA of the materials. Cellulose showed a rapid major weight loss between 250 and 350 °C due to dehydration and decomposition. CSs exhibited slow decomposition in a temperature range of 300–700 °C, which is typical for condensed carbonaceous materials. The produced CD demonstrated its major weight loss between 150 and 300 °C. This can be ascribed to the decomposition of the labile oxygen-containing moieties such as hydroxyl and carbonyl moieties.^46^ The weight loss of CDs in this temperature range corresponded to approximately 43% of the material. In further support of a successful carbonization, the char yields of CSs and CDs were 62 and 30%, respectively. This is clearly higher than the char yield of cellulose, which was 15% at 800 °C.

### Characterizations of Neat CA and CD-Modified CA Films

**Figure 2:**
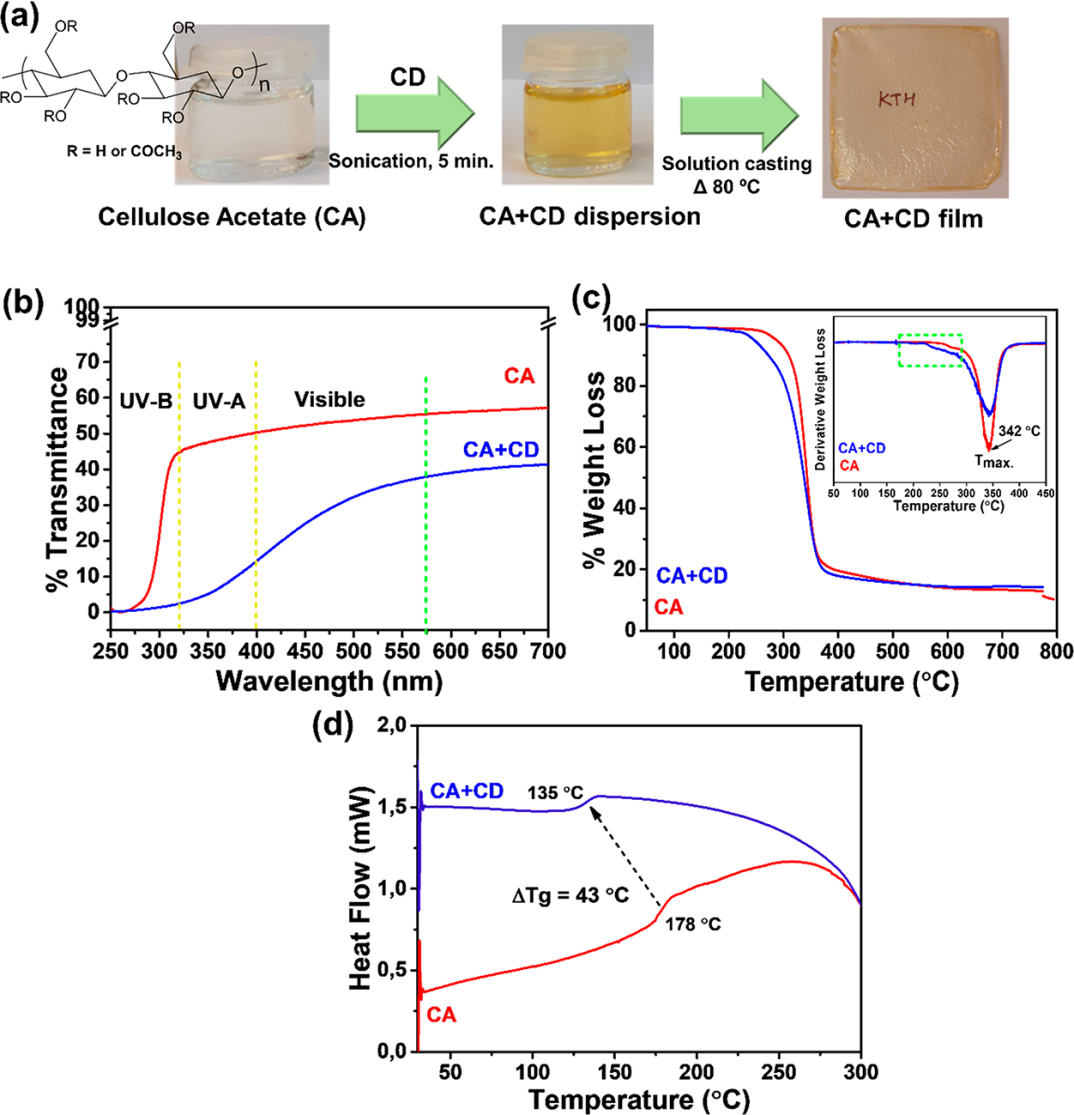
(a) Schematic presentation of the fabrication of CD-modified CA composite films (2.5 wt % loading of CDs); (b) UV–vis transmittance spectra, (c) TGA curves (DTG curves as the inset), and (d) DSC thermograms for CA and CA + CD films.

The produced neat CA film was colorless and transparent, while a light brownish color was introduced after addition of CD particles in the CA matrix as illustrated in Figure 2a. UV transmittance of CA and CA + CD films was evaluated in the wavelength range of 250–700 nm, Figure 2b. The analysis showed that the CA film transmitted a large part of the UV-A (315–400 nm) and visible light (400–700 nm). Noteworthy, the transmittance of UV-A and even visible light through CA + CD composite films was significantly lower. For instance, the UV transmittance was measured to be 53% for CA and 32% for CA + CD films at a wavelength of 500 nm. Simultaneously, the transmittance in the UV-A region was only 5% in the case of the CA + CD film confirming the absorption of most of the UV light at 370 nm. CA has no free electrons, which can contribute to high transmittance. CDs on the other hand contain free electrons because of the presence of π-conjugation and oxygen-rich functionalities, which absorb photons of the incident light.^47^ The incident light is therefore absorbed by the film and does not penetrate through it.

The TGA and DTG curves of the CA film and the CA + CD composite film are shown in Figure 2c. CA illustrated a one-step decomposition profile, which is related to the degradation of acetyl groups and cellulose main chain. The degradation on-set temperature of CA + CD was slightly lowered in comparison, which could be deduced to the low decomposition temperature of CDs and/or the catalyzing effect of CDs on the thermal degradation of CA. The *T*_max_ value, however, remained unaffected in comparison to the CA film. The DSC thermograms, Figure 2d, showed that the CD modification lowered the glass transition temperature (*T*_g_) by 43 °C compared with plain CA, which could further enhance the susceptibility of CA + CD to thermal degradation. Earlier it was reported that modification of CA with graphene and its derivatives led to higher mobility of the CA chains and decreased *T*_g_ values.^48^

### CD Triggered Degradation of CA under UV Irradiation in Air and Simulated Sea Water

The degradation of CA polymers with a high degree of acetylation proceeds slowly under natural and ambient conditions.^49^ The degradation and deacetylation process can differ greatly depending on factors such as solar radiation, humidity, temperature, and presence of microorganisms.^15,50^ Here, the potential of CD modification to trigger the degradation of CA was investigated under UV irradiation simulating the effect of sunlight and/or exposure to simulated sea water.

### Weight Loss and Visual Changes during Aging

**Figure 3:**
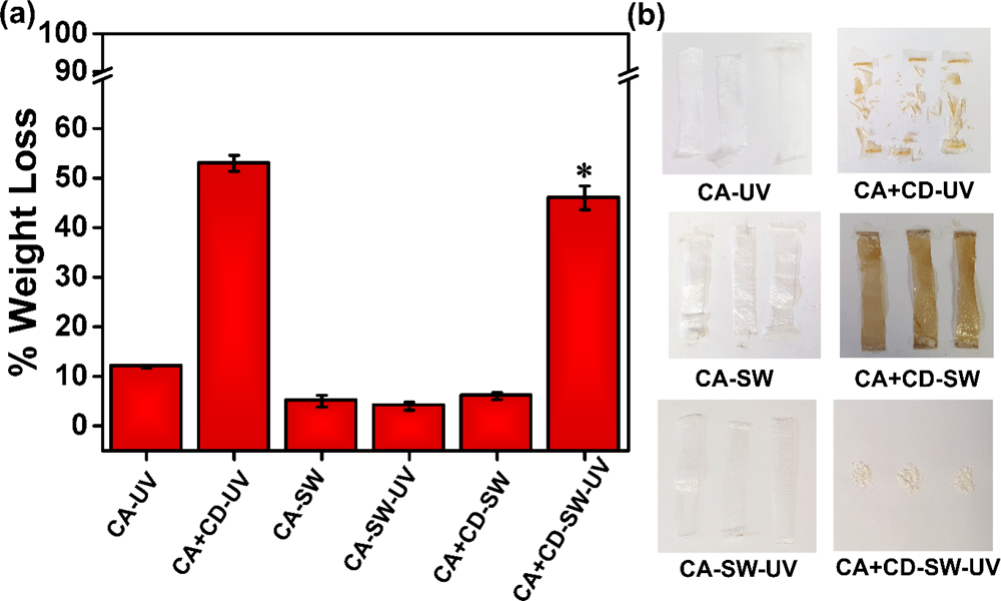
Weight loss of (a) CA films and CA + CD composite films under UV irradiation in air or in simulated sea water with or without UV irradiation (*mark indicates a sample with salt deposition, where the real weight loss was likely even larger). (b) Digital images of the aged samples.

The simplest way to evaluate the extent of degradation of polymers is to measure their overall weight loss during exposure.^51^ However, to know what is causing the weight loss and to ensure it is connected to real degradation, detailed characterization of the remaining polymer and released products is required.^3^ The extent of weight loss highly depends on the material formulation and environmental conditions. Figure 3a presents the weight loss of CA and CA + CD films after 30 days of UV irradiation in air (CA-UV and CA + CD-UV) and after 30 days aging in simulated sea water with (CA-SW-UV and CA + CD-SW-UV) or without (CA-SW and CA + CD-SW) UV irradiation. After the irradiation no changes occurred in the appearance of the CA-UV film, whereas the CA + CD-UV film had fragmented to small pieces. It was reported earlier that UV-A irradiation had no influence on the degradation rate of the CA polymer.^52^ In accordance, only negligible weight loss (∼2%) was observed after UV irradiation of CA fibers at <340 nm under vacuum conditions.^50^ In the present work, the CD modification significantly enhanced the degradation rate and weight loss of CA under UV irradiation. The weight loss of the neat CA film after 30 days of UV irradiation was approximately 12%, which can be compared to approximately five times higher weight loss, i.e., 53% for the CD-modified CA + CD film. The visual differences were also clear as demonstrated by the images in Figure 3b. Similar results were reported in the US Patent by Brodof and Hopkins, when 0.7% ultrafine TiO_2_ was incorporated as the photocatalyst in CA cigarette filters, which were subjected to humid and sunny climate of South Florida resulting in a weight loss of 65% within 6 months of exposure.^28^

The weight loss of CA-SW and CA + CD-SW after 30 days in simulated sea water without UV irradiation was low. For the films irradiated with UV in simulated sea water, the difference between CD-modified and plain CA films was instead significant. A weight loss of 4.3% was measured for CA-SW-UV, while the weight loss of CA + CD-SW-UV with CD modification was 43%. This clearly showed the ability of CD modification in combination with UV irradiation to trigger the simulated open environment degradation process. The weight loss of CA + CD-SW-UV in simulated sea water could even have been slightly underestimated due to salt deposition on the surface. Although the degradation temperature during UV irradiation was slightly higher due to the heating caused by the UV lamps, the almost identical weight loss for CA-SW and CA-SW-UV indicates that the small temperature difference did not have a significant effect on the degradation process. An interesting observation was made by comparing the images of the CA + CD samples after UV irradiation in air and sea water. Both samples had fragmented but the sample aged in water additionally lost the brownish color. This could indicate different aging processes in air and simulated sea water. Since the films aged in water without UV irradiation were still brown and the aging solutions were transparent, the loss of color could not be explained by simple migration of CDs to the aqueous phase.

### Changes in Molar Mass during Aging

To evaluate the reason behind the observed weight loss and fragmentation of the samples, SEC analysis was performed to determine the molar mass changes for CA and CA + CD during aging under different conditions. It was shown that UV irradiation significantly decreased the molar mass of both CA-UV and CA + CD-UV in air and CA + CD-SW-UV in simulated sea water (Table 1). Under air conditions the weight average molar mass (*M*_w_) decreased 78% for CA-UV and 83% for CA + CD-UV in comparison to neat CA before aging. Although there was no large difference in the molar mass decrease measured for CA-UV and CA + CD-UV in air, the significantly larger weight loss of >50% of CA + CD-UV demonstrates clearly the higher degree of degradation that has led to fragmentation and formation of gaseous products (Figure 3). This is further supported by the decreased Đ for CA + CD-UV.

**Table 1:** Average Molar Mass and Dispersity of CA and CA + CD before and after Aging under Different Conditions

sample	elution vol. (mL)	*M*_n_ (g/mol)	*M*_w_ (g/mol)	*M*_z_ (g/mol)	*Đ*
CA	16.50	12,000	65,000	140,000	4.80
CA-UV	18.42	3300	14,000	35,000	4.32
CA + CD	16.45	14,000	62,000	140,000	4.42
CA + CD-UV	18.90	3300	11,000	26,000	3.34
CA-SW	16.45	12,000	66,000	170,000	5.80
CA-SW-UV	16.50	14,000	62,000	130,000	4.47
CA + CD-SW	16.43	21,000	68,000	150,000	3.30
CA + CD-SW-UV	18.46	6600	15,000	33,000	2.31

After aging under simulated sea water conditions without UV irradiation, the molar mass of CA-SW was still comparable with the original molar mass of neat CA. In the case of CA + CD-SW aged in sea water without UV irradiation, the *M*_w_ and *M*_z_ values were close to the original values but *M*_n_ had increased. This is a typical first sign of the degradation process, where some lower molar mass products have started migrating from the films. Initially, the *M*_n_ value will increase as it is highly influenced by the low molar mass fraction. At the same time, *M*_w_ and *M*_z_, which are more influenced by the chains with higher molar masses, are left unaffected during initial stages of degradation.

The addition of CDs clearly triggered the degradation of CA in sea water under UV irradiation. The M_w_ of CA-SW-UV decreased by only 5%, whereas the corresponding decrement was 77% for CA + CD-SW-UV. Altogether the different molar mass averages of CA-SW-UV were quite close to those of the original CA and CA-SW. The different average molar masses of CA + CD-SW-UV on the other hand decreased to almost the same degree as the average molar masses of CA + CD-UV irradiated in air, demonstrating the great potential of CD modification to even accelerate the degradation of CA in sea water. This correlated well with the large and relatively similar weight losses for CA + CD-UV and CA + CD-SW-UV.

### Spectroscopic Characterization of Aging-Induced Changes

**Figure 4:**
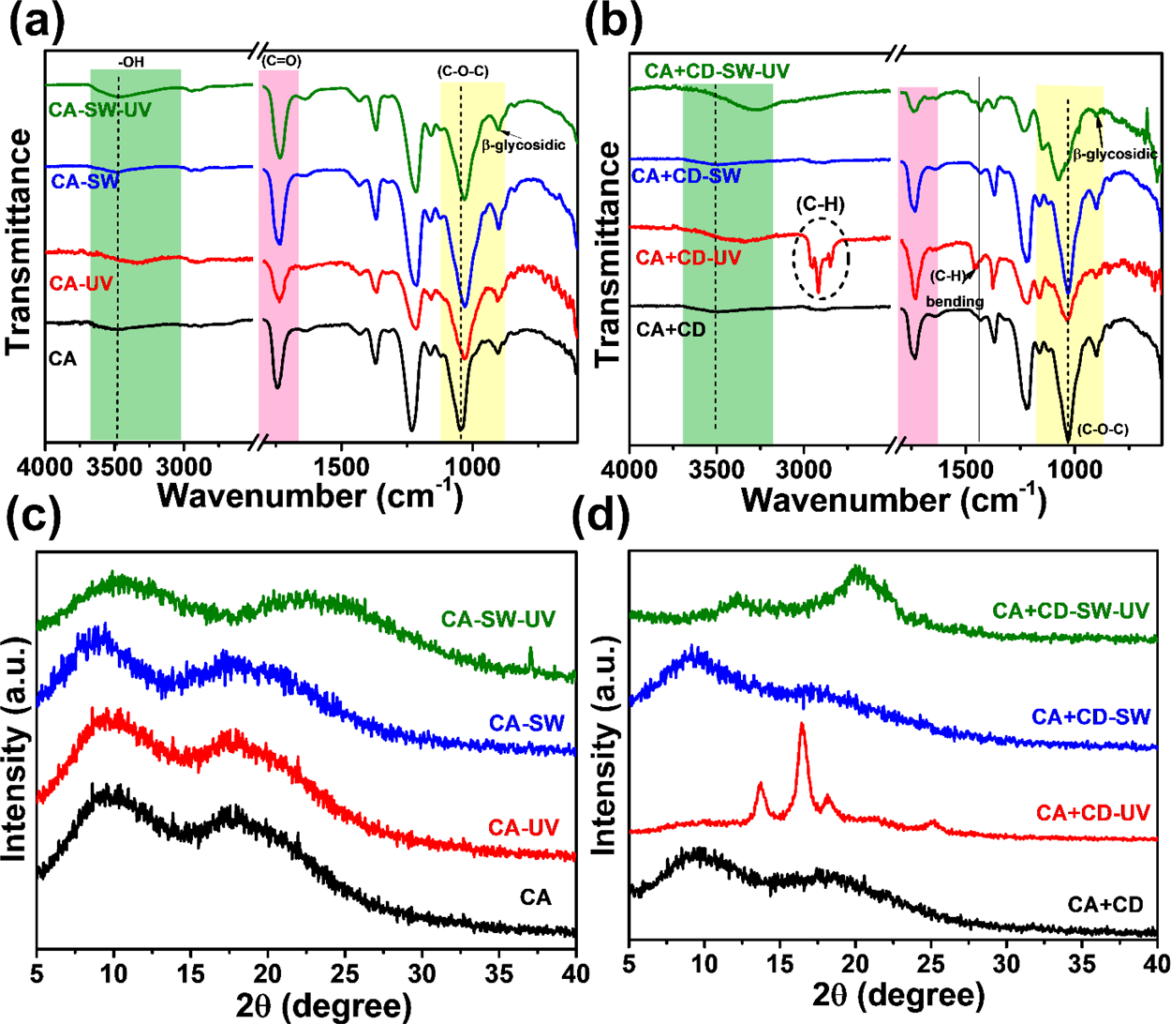
Structural characterization of degraded samples in different environments: (a and b) FTIR and (c and d) powder XRD analysis of degraded CA and CA + CD films.

FTIR was utilized to map the changes in the functional groups due to the aging of CA and CA + CD films (Figure 4a,b). The FTIR spectra remained unchanged in most cases after the aging of CA films with or without UV irradiation. A minor shifting of the C–O–C band could be observed for all aged CA samples. In addition, a small increase in the −OH absorption band could be observed for CA-SW-UV, indicating possibly some deacetylation leading to the increased amount of hydroxyl groups. In correlation with the larger weight loss, more significant chemical changes were observed in the UV-irradiated CA + CD samples, CA + CD-UV and CA + CD-SW-UV. In the case of CA + CD-UV subjected to direct UV exposure, Figure 4b, the appearance of the sharp −C–H absorption band is prominent and assumed to correlate with the released acetic acid on the surface of the films. In the case of CA + CD-SW-UV, the FTIR band at ∼3300 cm^–1^ increased in intensity and shifted to a lower wavenumber, indicating the higher amount of hydroxyl functionalities formed by deacetylation and generation of intermolecular and intramolecular hydrogen bonds. This suggests at least partial transformation from CA to the cellulose-type structure.^53,54^ This is further supported by the decreased intensity of the carbonyl absorption band. During aging under aqueous conditions, the degraded products, like acetic acid, can migrate to the aqueous phase. No significant structural differences were observed in the case of plain CA films after UV irradiation, Figure 4a, which correlated with the weight loss data. In the case of CA-SW-UV, a small increase in the intensity of hydroxyl absorbance was observed, which could indicate some deacetylation taking place. In agreement with the weight loss results, without UV exposure, none of the films exhibited significant changes in the functional groups during the aging period.

XRD diffraction patterns of CA and CA + CD films are displayed in Figure 4c,d. The XRD patterns of neat CA and CA + CD films before aging contained two broad reflections located at 10 and 18°. During aging, the original diffraction pattern remained almost unchanged for CA-UV and CA-SW, while some further broadening of the reflections and shift toward higher 2θ were observed for CA-SW-UV, Figure 4c. This agrees with the somewhat larger weight loss observed for CA-SW-UV, which in turn influenced the crystalline part of the polymer. More remarkable changes were observed after UV irradiation of CA + CD composites, especially in air, Figure 4d. CA + CD-UV displayed clear diffraction patterns of cellulose, strongly indicating deacetylation during the UV irradiation leading to partial regeneration of cellulose fibers and the cellulose crystal structure.^13^ In the case of CA + CD-SW-UV, the reflection had started to sharpen, possibly indicating that regeneration of cellulose fibers had been initiated.

**Figure 5:**
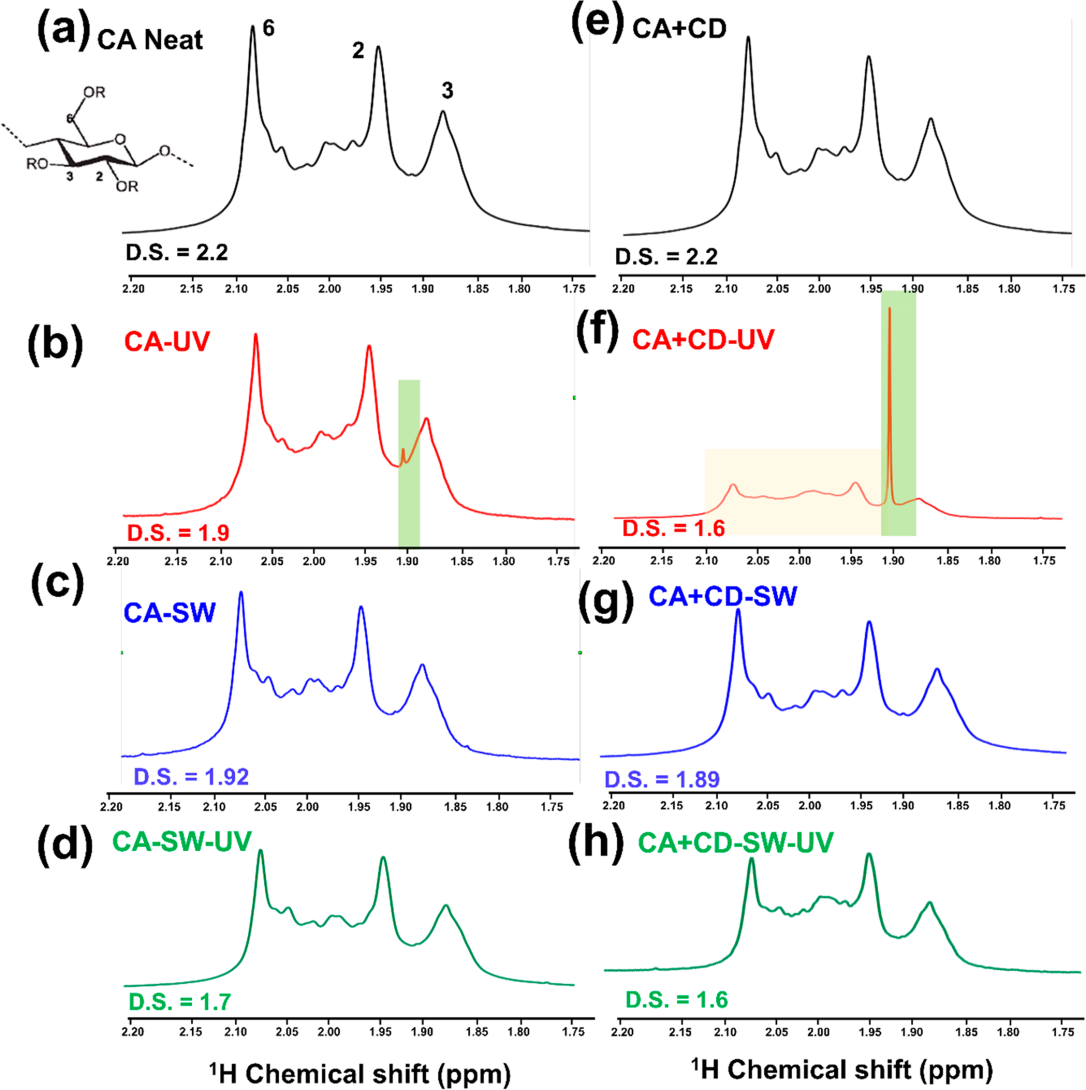
^1^H NMR spectra of CA and its composite films in DMSO-d6 before and after UV irradiation for 30 days.

To further understand the degradation mechanism and to support the chemical changes observed by FTIR spectroscopy, the CA and CA + CD films before and after aging were analyzed by ^1^H NMR. In the spectra of original CA and CA + CD films before UV irradiation (Figure 5a,e), three peaks at 1.6–2.3 ppm were observed corresponding to the acetyl substitution at 2, 3, or 6 position in AGU of CA.^55−57^ After the UV irradiation in air, a sharp strong peak appeared at 1.91 ppm in the spectra of CA + CD-UV (Figure 5b,f). This peak corresponds to the presence of acetic acid, which was also observed in the FTIR spectrum of CA + CD-UV. A signal with lower intensity of the same peak was also observed in the ^1^H NMR spectrum of CA-UV. Similar observation has been seen after the addition of organic photosensitizers to the CA polymer.^24^ Significant deacetylation, thus, took place during the UV irradiation of CA + CD-UV and to a lower degree for CA-UV. In agreement, the DS^24^ calculated from the ^1^H NMR spectra decreased from 2.2 for CA and CA + CD to 1.9 for CA-UV and 1.6 for CA + CD-UV.

In simulated sea water without UV irradiation, the DS of CA-SW and CA + CD-SW decreased very moderately to 1.9 (Figure 5c,g). When the films were instead aged in water with UV irradiation, the DS decreased to 1.7 for CA-SW-UV and even more significantly to 1.6 for CA + CD-SW-UV (Figure 5d,h). This correlated with the changes observed in the FTIR spectrum and the greater weight loss of CA + CD-SW-UV. The liberated acetic acid from the films aged in simulated sea water was likely released to the aqueous phase and the peak corresponding to acetic acid was not detected in the NMR spectra. This was supported by the slight decrease in the pH of the aqueous phase (∼5.8 ± 0.6) in the case of CA + CD-SW-UV.

### Thermal Properties of Age-Induced Changes

**Figure 6:**
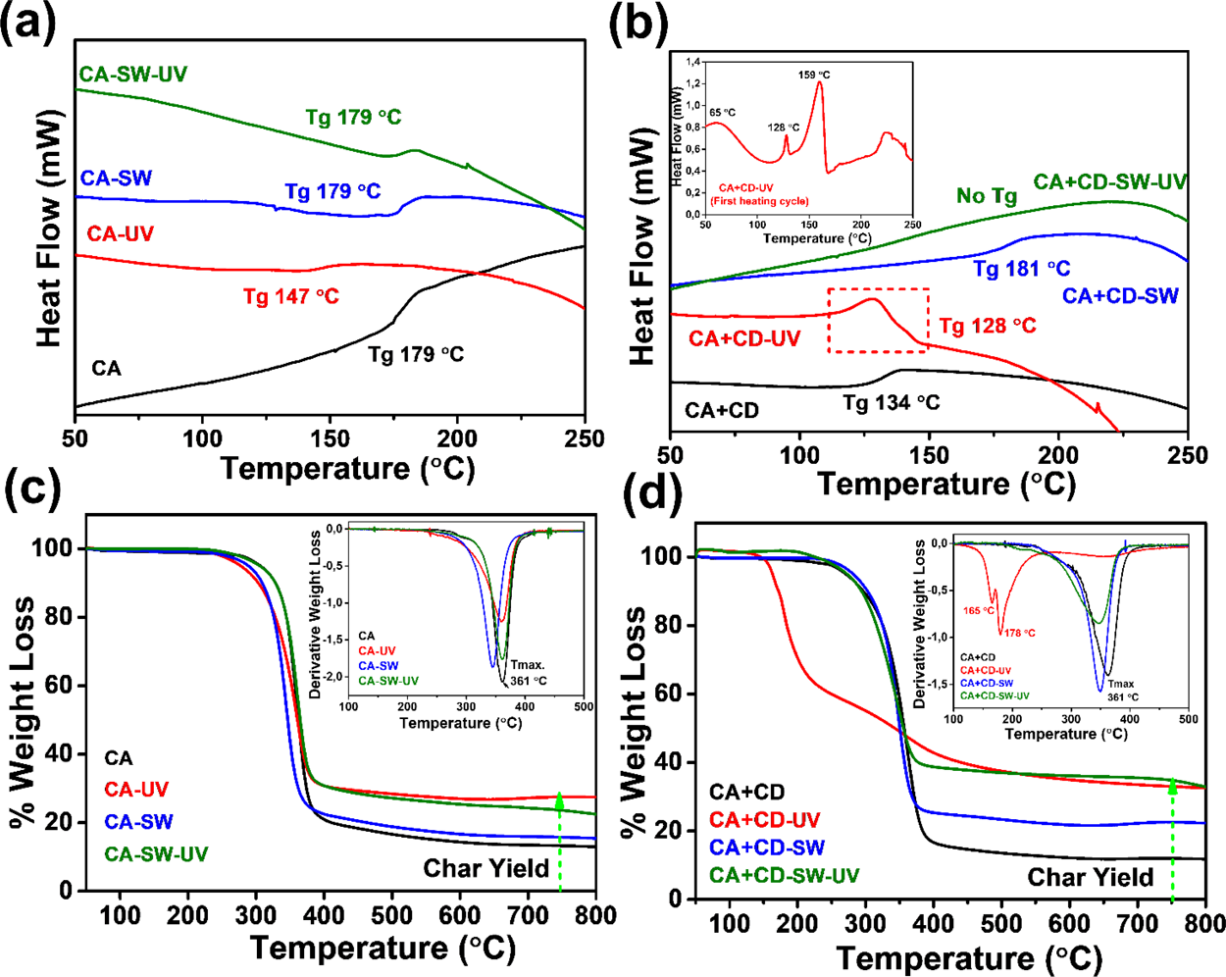
Thermal characterization of the samples aged in different environments: (a) DSC thermograms (inset from first heating) and (b) TGA of the degraded samples.

The effect of aging on the *T*_g_ of the films was evaluated by DSC. Aging of CA in simulated sea water with or without UV irradiation did not significantly affect the *T*_g_ value of CA-SW and CA-SW-UV. However, when CA was UV-irradiated in air (CA-UV) the *T*_g_ value dropped significantly to 147 °C from 179 °C for CA, Figure 6a, which correlates with the greatly reduced molar mass. In the case of CA + CD, DSC analysis indicated significant changes in the materials. For CA + CD-UV, Figure 6b, the *T*_g_ additionally decreased to 128 °C. To further elucidate the effect of aging and the nature of species generated, the first heating cycle (30–300 °C) was also evaluated, Figure 6b, inset. Here, the broad initial endotherm (maxima at 65 °C) was observed that could be attributed to the evaporation of acetic acid from the sample.^58^ The two sharp peaks at 128 and 159 °C might be due to, for example, formation of oligosaccharide units, acetylated glucose units, or destruction of pyranose rings.^22^ The aging in simulated sea water under UV irradiation (CA + CD-SW-UV) instead increased the *T*_g_ temperature, which can be connected to the deacetylation and partial regeneration of the cellulose structure.^59,60^ Without UV treatment no significant changes were observed.

The TGA of original and aged samples showed the one-step degradation profile due to degradation of the cellulose structure at 361 °C, Figure 6c. The exception was the highly degraded CA + CD-UV sample, which degraded in two steps. In the case of CA samples, the char yield increased for the samples that were UV-irradiated and could be connected to the deacetylations, which might increase the carbonization during the thermal treatment. The presence of acetic acid in the CA-UV sample could additionally catalyze the carbonization reaction. For CA + CD samples, the char yield increased for all samples and it was higher compared with the corresponding CA samples. This can be explained by the higher degree of degradation and deacetylation in combination with the ability of CDs to increase the char formation. The DTG curve of CA + CD-UV, (Figure 6d, inset) illustrated two broad degradation profiles at lower temperature maxima at 165 and 178 °C, which could be associated with the high degree of degradation and presence of acetic acid and other low molar mass compounds.

### Morphological Analysis of Aging-Induced Changes

**Figure 7:**
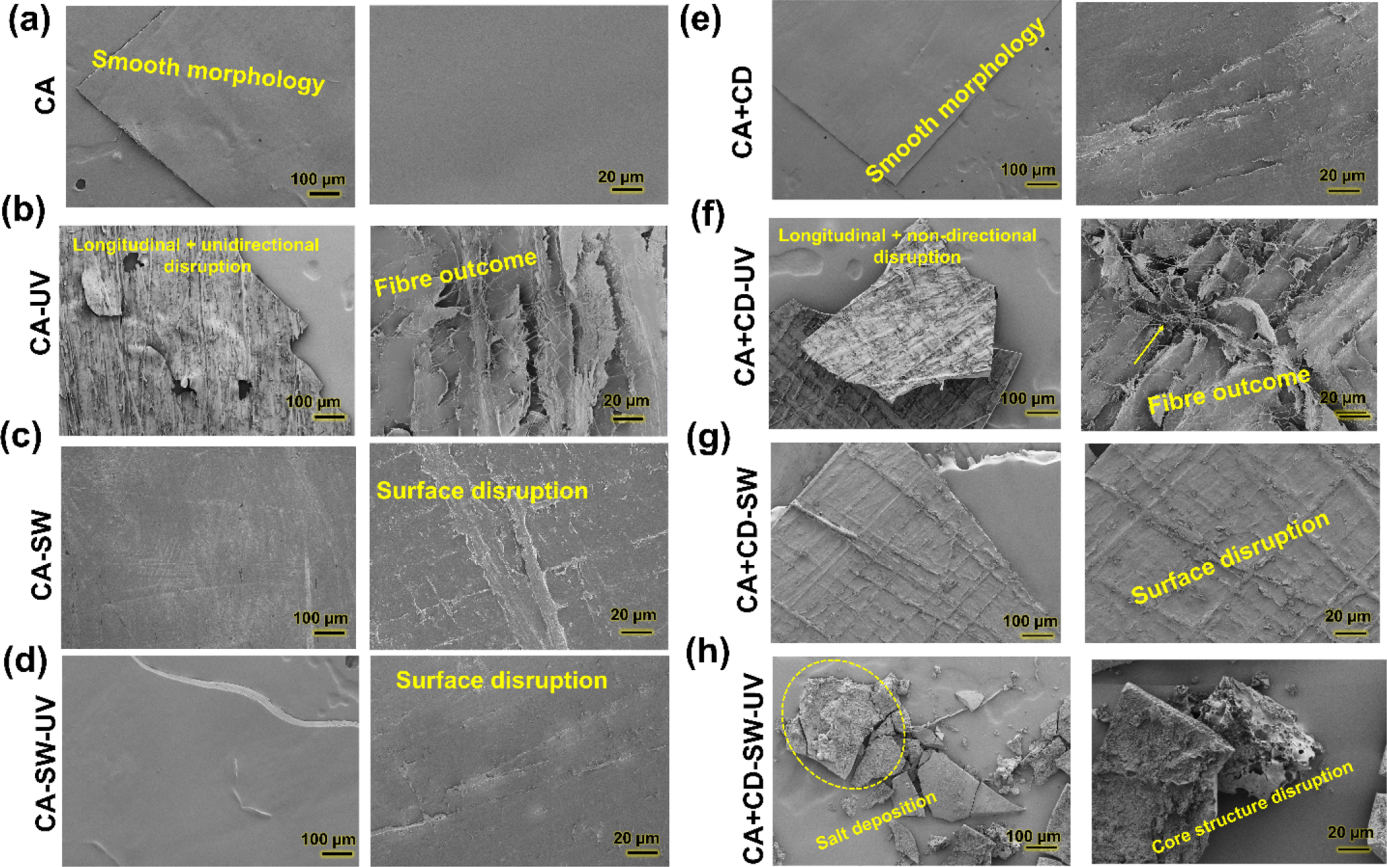
SEM images of CA films and CA + CD composite films before and after aging under different environment conditions.

To analyze the surface changes of the films caused by aging, the films were imaged by SEM (Figure 7). The original CA and CA + CD films had a smooth surface morphology with some surface scratches, Figure 7a,e. After UV irradiation of the films in air, Figure 7b,f, severe surface disruption could be noticed on the films along with the formation or outcome of fibrous structures that may be due to deacetylation and regeneration of cellulose fibers. For CA-SW, CA + CD-SW, and CA-SW-UV, Figure 7c,g,d, some surface disruption was observed compared with the films before aging. The addition of CDs in the film in combination with UV irradiation (CA + CD-SW-UV) gave rise to fragmentation of the films (Figure 7h). In addition, the surface and core structure had been disrupted. Some salt deposition was also observed as thorough washing was not done to avoid any further effects on the surface.

### Evaluation of the Degradation Products Released to the Simulated Sea Water

**Figure 8:**
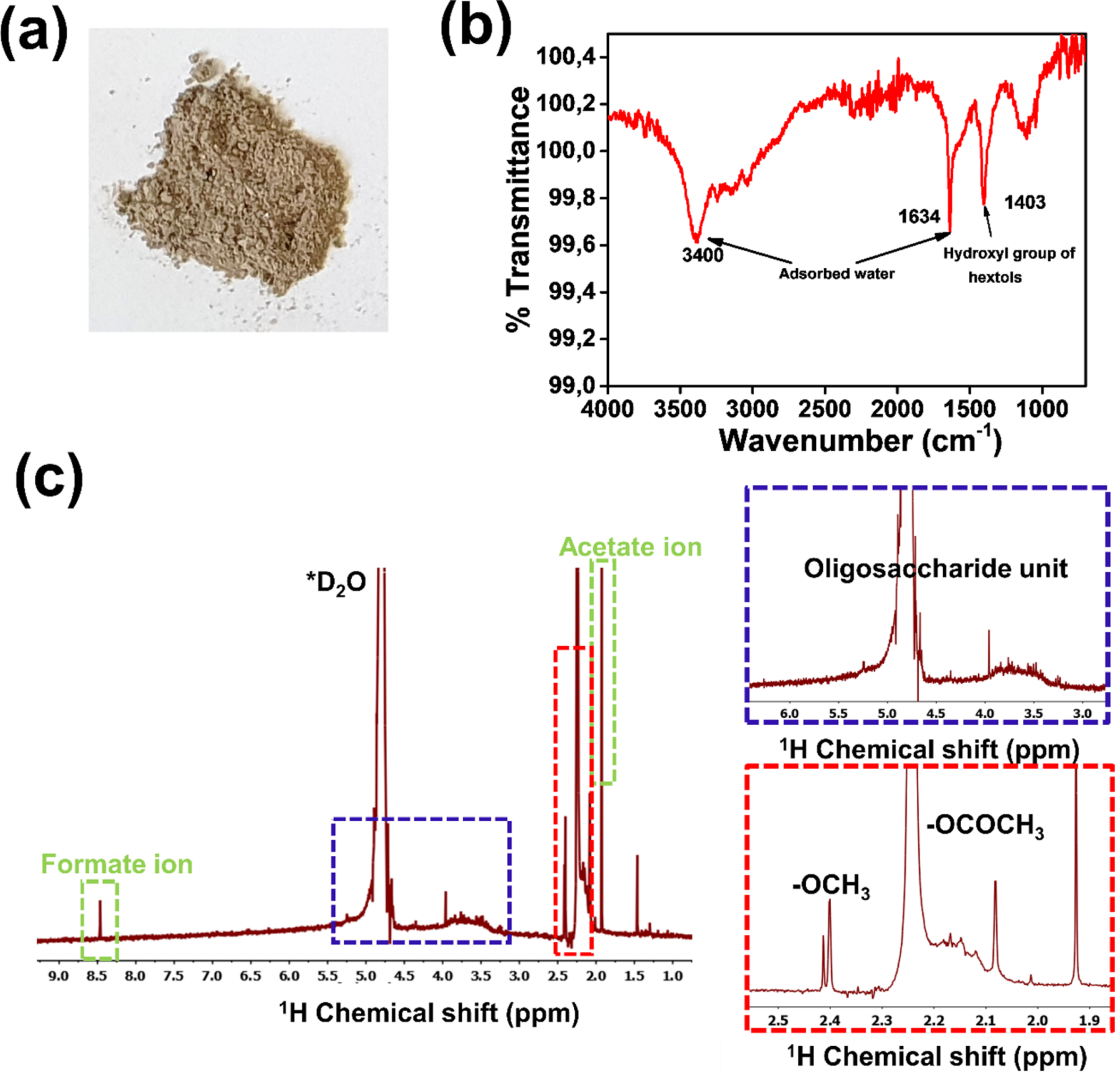
(a) Dried mixture of water-soluble degradation products and salts after aging of CA + CD-SW-UV in simulated sea water under UV irradiation. (b) FTIR and (b) ^1^H NMR spectrum of the degradation products. The ^1^H NMR spectrum was recorded in D_2_O.

The sea water after aging of the most degraded material, CA + CD-SW-UV, was evaporated to identify the leached-out materials. The leached-out material had a brownish color and consisted of a combination of compounds originating from degradation of cellulose acetate and different salts used for preparation of simulated sea water (Figure 8a). FTIR spectra of the recovered products is shown in Figure 8b. There are distinct absorption peaks at 1415 cm^–1^ possibly attributed to the hydroxyl group of hextols and at 1650 and 3400 cm^–1^ assigned to adsorbed water and hydroxyl functionalities.^61^ For further characterization of the leached-out material, ^1^H NMR was performed. In the spectrum, Figure 8c, the prominent peaks were assigned to the presence of formate ions (8.5 ppm), mixtures of oligosaccharide units (5.4–3.3 ppm), methoxy or acetyl groups (2.80–2.00 ppm), and acetate ions (1.90 ppm).

### Photocatalytic Generation of H_2_O_2_ by CD Particles

**Figure 9:**
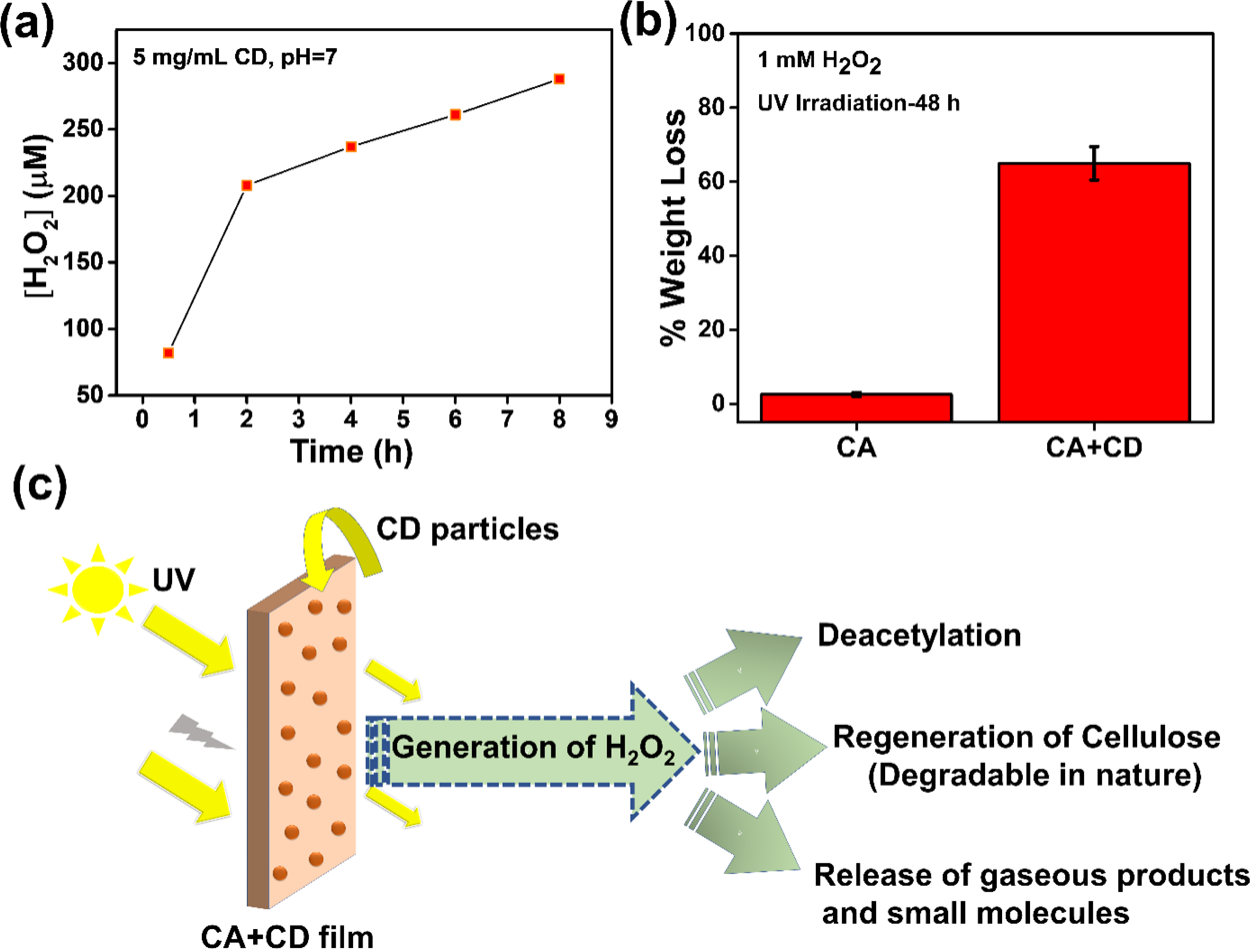
(a) Photoproduction of H_2_O_2_ under simulated sunlight at pH 7. (b) Weight loss determination after addition of H_2_O_2_ (1 Mm) externally into the water system followed by UV irradiation for 48 h. (c) Plausible mechanism of degradation of the CA + CD film under the effect of UV irradiation simulating sunlight.

It has been reported that carbon-based nanomaterials can efficiently photocatalyze millimolar level generation of H_2_O_2_ under simulated sunlight in a few hours.^62^ Upon UV irradiation, the CD particles can, thus, in the presence of oxygen and water, catalyze the formation of H_2_O_2_ through radical species. We confirmed the formation of H_2_O_2_ in the presence of CDs in water by the DMP assay as shown in Figure 9a. The results show that CD particles were photocatalytically active under simulated sunlight conditions and generated micromolar levels of H_2_O_2_ (∼270 μM during 8 h of UV irradiation). Similar photocatalytic activity for H_2_O_2_ synthesis under visible light has been observed in the case of TiO_2_, graphene-based nanomaterials, lignin, carbon nitride, and their related composites.^63−65^

It is well established that UV radiation can initiate photo-oxidation of most polymers. The photo-oxidation can proceed via the radical chain mechanism that results in bond cleavage and a decrease in the molecular weight.^66^ This suggested that the incorporation of CD particles into the CA matrix in catalytic amounts could initiate a faster photodegradation process via the formation of radical species. To evaluate this hypothesis, H_2_O_2_ (1 mM) was added externally into the aqueous solution with solid CA or CA + CD films. After 48 h of irradiation, the neat CA film illustrated a low weight loss of ∼2.5%, whereas the CA + CD film displayed a weight loss of more than ∼65% with the content of remaining fragments, as shown in Figure 9b. The degradation products that migrated out into the aqueous phase were similar to the leached-out compounds from CA + CD-SW-UV after UV irradiation without addition of H_2_O_2_ as shown in Figure 8.

Thus, a plausible mechanism is the initiation of free radicals from O_2_, H_2_O, and H_2_O_2_ (self-generated) catalyzed by CDs under UV irradiation. This could lead to oxidative fragmentation of CA, regeneration of cellulose fibers, and formation of acetic acid due to deacetylation and formation of gaseous products, such as CO_2_, CO, and water. In the water system, soluble small molecules (e.g., acetic acid) and oligosaccharides were leached out from the polymer core.

## Conclusions

CDs, produced by HTC and oxidation of cellulose, were demonstrated as effective photocatalysts that are able to trigger the degradation of CA under simulated sunlight (UV-A irradiation). Incorporation of CDs leads to generation of H_2_O_2_, which is expected to initiate free radicals under UV irradiation. This catalyzed the deacetylation process, leading to the formation of small molecules and regenerated cellulose fibers. As native cellulose is inherently degradable in natural environments, this triggered deacetylation is expected to render the material completely biodegradable. New insights into photocatalytic degradation of CA in air and simulated sea water were gained by careful mapping of the degradation process by following changes in the weight loss, molecular weight, chemical structure, thermal properties, and morphology and release of degradation products. The potential of CDs as green photocatalysts for plastic degradation deserves further attention.
